# Regulation of miRNAs as new tool for cutaneous vitality lesions demonstration in ligature marks in deaths by hanging

**DOI:** 10.1038/s41598-019-56682-7

**Published:** 2019-12-27

**Authors:** Margherita Neri, Matteo Fabbri, Stefano D’Errico, Marco Di Paolo, Paola Frati, Rosa Maria Gaudio, Raffaele La Russa, Aniello Maiese, Matteo Marti, Enrica Pinchi, Emanuela Turillazzi, Vittorio Fineschi

**Affiliations:** 1 Department of Morphology, Experimental Medicine and Surgery, University of Ferrara, Ferrara, Italy; 2 Department of Legal Medicine, Azienda Ospedaliera Universitaria Sant’Andrea, via di Grottarossa, Rome, Italy; 3 Section of Legal Medicine, Department of Surgical Pathology, Medical, Molecular and Critical Area, University of Pisa, Pisa, Italy; 4 Department of Anatomical, Histological, Forensic and Orthopaedic Sciences, Sapienza University of Rome, Rome, Italy; 5 IRCSS Neuromed Mediterranean Neurological Institute, Pozzilli, Italy

**Keywords:** Diagnostic markers, Genetics research

## Abstract

This study aims to demonstrate that the application of miRNA expression in forensic pathology, in cases of hanging, applying the method on skin samples. The proposed investigative protocol allowed us to highlight a different miRNA expression in the skin ligature marks of subjects who died by hanging compared to healthy skin control samples. The results obtained showed an increase in the expression of miRNAs recognized as regulators of the inflammatory response in skin lesions such as miR125a-5p and miR125b-5p. Furthermore, overexpression of additional miRNAs – miR214a-3p, miR128-3p, miR130a-3p, and miR92a-3p – with anti-inflammatory activity was highlighted. It was possible to document a statistical significance to control skin samples only for miR103a-3p (p < 0.05), miR214-3p and miR92a-3p (p < 0.01) The upregulation of miR222-3p and miR150-5p, respectively related to mast-cell activation and neutrophils after the application of traumatic stimuli supports the immunohistochemical data showed in literature. The diagnostic accuracy of miRNAs could expand the range of diagnostic tools available in the assessment of the vitality of a lesion.

## Introduction

miRNAs are small, endogenous, non-coding molecules that act as master regulators of cellular processes, primarily at the post-transcriptional level. They regulate gene translation by attenuating protein translation through the promotion of their mRNA degradation^1,2^. miRNAs target more than one hundred mRNAs, thus potentially affecting a great number of pathophysiological pathways^3–5^.

When the skin, like many other organs, is injured, a complex, highly-orchestrated healing process starts, requiring the interplay and crosstalk of a multitude of cells and mediators^6,7^. There are different, overlapping and successive phases, including the hemostasis/inflammatory, proliferative, and remodeling phases, overlap and follow one another. Each has a histological and biomolecular imprint that has been widely investigated; these are collectively termed ‘vitality’, and relate to whether or not the victim was alive at the time the trauma was sustained^8–12^.

However, wound age evaluation is one of the hardest challenges for the forensic pathologist when asked to establish the vitality of a skin lesion since, especially at the very beginning of the healing process, traditional histological and immunohistochemical examinations may not provide solid objective evidence^13^. Consequently, research into the numerous biological substances involved in the process of wound repair has been carried out over the years to identify increasingly reliable biomarkers even in the very early stages of the healing process^14^ and advanced techniques have been applied to generate data with enhanced accuracy and objectivity^15^.

Since miRNAs play a pivotal role in regulating the expression of key proteins that control the complex inflammatory response^16–19^ and since, after wounding, the mRNA levels of cytokines and enzymes typically change sooner than protein levels and the histomorphology^20–23^, we proceeded to investigate whether the expression of some selected miRNAs was modified in ligature marks (patterned abrasion caused by ligature material) in death by hanging. At the same time, we acknowledged that gross and histological examination of these marks may sometimes be unreliable and may mislead the forensic pathologist into concluding as to whether they are due to hanging or post-mortem suspension of the body^24^.

In this study we investigate the expression of a panel of miRNAs in skin specimens in autopsy cases of death due to hanging, to clarify and to discuss their significance in assessing whether hanging marks and signs occurred before or after the death of the victim.

## Results

### Expression of the selected miRNAs

**Figure 1:**
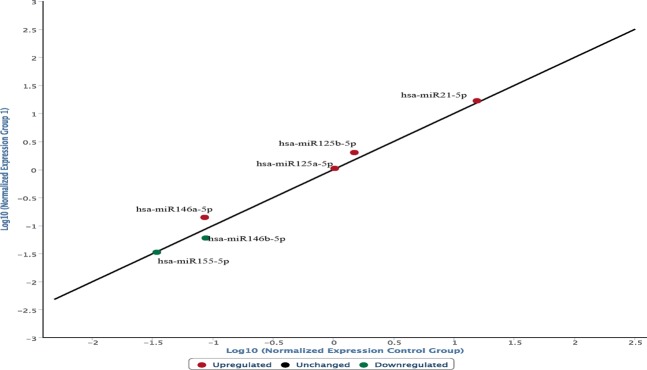
microRNAs expressed in frozen samples of skin from the hanging ligature marks are compared to control group skin samples. The straight black line corresponds to the average of the miRNA values obtained from the control skin samples, first normalized and then expressed as a logarithmic function on the abscissa axis, while the microRNA values of the ligature marked skin are shown on the ordinate axis.

Graphs were constructed highlighting the expression of the selected miRNAs in the ligature marked skin samples compared to the non-injured skin samples. In the following graph, microRNAs expressed in frozen samples of skin from the hanging ligature marks are compared to control group skin samples (Fig. 1). The graph is characterized by the presence of a straight black line corresponding to the average of the miRNA values obtained from the control skin samples, first normalized and then expressed as a logarithmic function on the abscissa axis, while the microRNA values of the ligature marked skin are shown on the ordinate axis.

The frozen skin samples from the ligature marks show a different expression of the microRNAs involved in the regulation of the cutaneous inflammatory phase:

- miR146a-5p, miR125a-5p, miR125b-5p, miR21-5p, identified in the graph with a red dot, are over-expressed;
- miR146b-5p e miR155-5p, identified in the graph with a green dot, are underexpressed.

**Figure 2:**
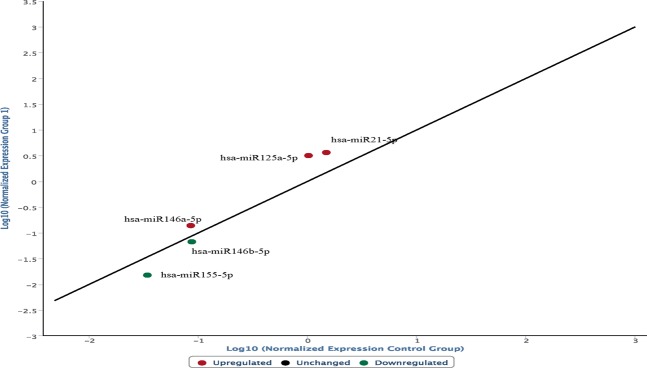
Modifications in the expression of the microRNAs from ligature mark samples embedded in paraffin compared to the frozen sample group. An increased expression of miR-146a-5p, miR125a-5p, miR21 and a hypo-expression of miR155-5p and miR146b-5p were observed in the ligature marks investigated, regardless of whether the samples were frozen or embedded in paraffin.

The following graph shows the modifications in the expression of the same microRNAs from ligature mark samples embedded in paraffin compared to the frozen sample group (Fig. 2).

In conclusion, an increased expression of miR-146a-5p, miR125a-5p, miR125b-5p, miR21 and a hypo-expression of miR155-5p and miR146b-5p were observed in the ligature marks investigated, regardless of whether the samples were frozen or embedded in paraffin.

We decided not to proceed further in the investigation of underexpressed miRNAs since their down-regulation could be related both to an actual diminished expression and to a degradation of the RNA molecule due to the action of formalin and paraffin.

**Figure 3:**
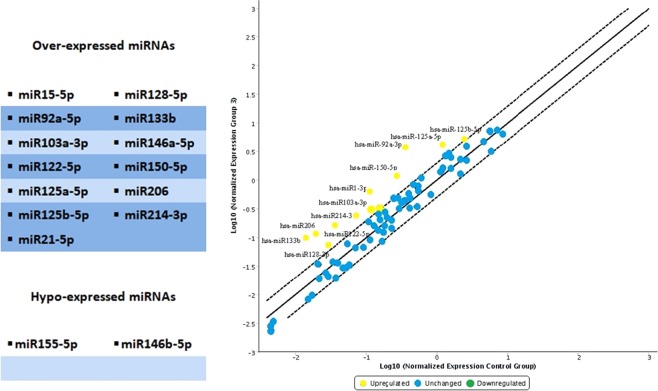
The degree of microRNA expression in the ligature marked skin was compared with the controls considered in the norm in a range of values between the two dashed lines. In this way it was possible to highlight that the most overexpressed miRNAs in the ligature marked skin samples were: miR128-5p, miR214-3p, miR133b, miR206, miR122-5p, miR103a-3p, miR150-5p, miR92a-5p. miRNAs that regulate the cutaneous inflammatory response (miR125a-5p and miR125b-5p) were also overexpressed.

The analysis of the overexpressed miRNAs in the hanging ligature marks was extended and the following graph was derived (Fig. 3). The degree of microRNA expression in the ligature marked skin was compared with the controls considered in the norm in a range of values between the two dashed lines.

In this way, it was possible to highlight that the most overexpressed miRNAs in the ligature marked skin samples were: miR128-5p, miR214-3p, miR133b, miR206, miR122-5p, miR103a-3p, miR150-5p, miR92a-5p. Furthermore, those miRNAs that regulate the cutaneous inflammatory response, i.e., miR125a-5p and miR125b-5p, were also overexpressed^18,25,26^.

**Figure 4:**
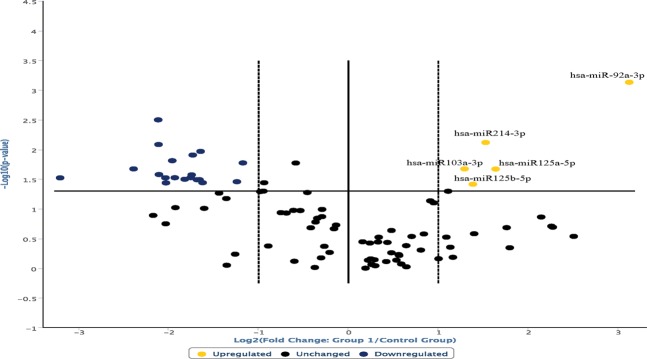
Overexpression of miR125a-5p, miR125b-5p and miR103a-3p observed in ligature marked skin samples was statistically significant (p < 0.05).

Data were elaborated through the Student’s test, allowing us to highlight that the overexpression of miR125a-5p, miR125b-5p and miR103a-3p observed in ligature marked skin samples was statistically significant (Fig. 4). The data obtained, repeated in numerical values of 2^−ΔCT^ (second order variation of the values expressed in CT obtained from the analysis of the sample through RT-PCR), show a statistically significant increase in miR125a-5p and miR125b-5p (p < 0.05) (Table 1).

**Table 1:** Statistical analysis: the data obtained are repeated in numerical values of 2^−ΔCT^ (second order variation of the values expressed in CT obtained from the analysis of the sample through RT-PCR).

miRNA	2^ΔCt		p-value
	Skin mark	Skin healthy	
hsa-miR146a-5p	0,138	0,085	0,2900
hsa-miR125a-5p	3,169	1,019	0,0210
hsa-miR125b-5p	3,629	1,483	0,0210
hsa-miR92a-3p	2,616	0,299	0,000
hsa-miR214a-3p	0,177	0,061	0,007
hsa-miR103a-3p	0,115	0,316	0,040

**Figure 5:**
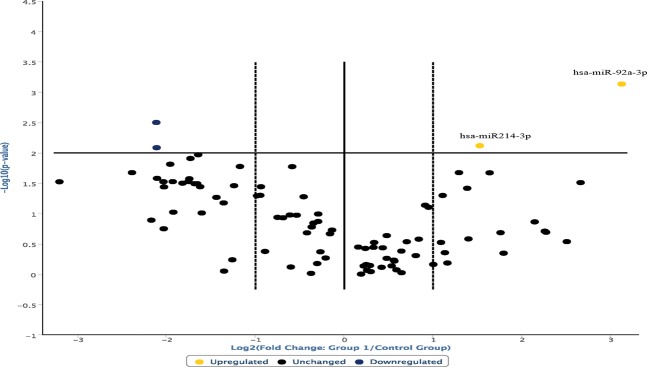
Analysis of the expression profiles of miR214-3p and miR92a-3p demonstrated an up-regulation in the group of subjects who died by hanging compared to controls. Statistically significant increase for miR103a-3p (p < 0.05), miR214-3p and miR92a-3p (p < 0.01).

The analysis of the expression profiles of miR214-3p and miR92a-3p also demonstrated an up-regulation in the group of subjects who died by hanging compared to controls (Fig. 5). Statistical analysis of the results through the Student test showed a statistically significant increase for miR103a-3p (p < 0.05), miR214-3p and miR92a-3p (p < 0.01) (Table 1).

**Figure 6:**
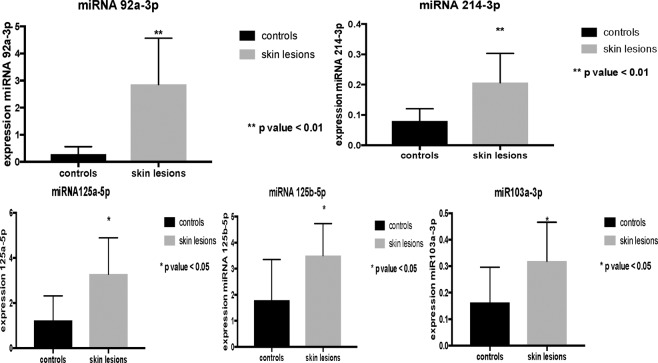
Whiskers plot about the statistical significance of the data obtained to further characterize the expression profiles with respect to the control skin samples as well as to highlight a different significance in the context of the up-regulation microRNA panel. Statistical analysis of the results through the Student test showed a statistically significant increase for miR103a-3p (p < 0.05), miR214-3p and miR92a-3p (p < 0.01).

The evaluation of the statistical significance of the data obtained allowed us to further characterize the expression profiles compared with the control skin samples as well as to highlight a different significance in the context of the up-regulation microRNA panel (Fig. 6).

Statistical analysis of the results through the Student test showed a statistically significant increase for miR103a-3p (p < 0.05), miR214-3p and miR92a-3p (p < 0.01) (Table 1).

In the search for a possible correlation between miRNA expression and immunohistochemical findings previously reported in ligature marks^24^, i.e., the positivity for tryptase, IL-15 and CD15, miR222-5p, involved in the activation of mast cells, and miR150-5p, altered in neutrophils after traumatic tissue events, were investigated^27^. According to, our histologic result demonstrated the evidence for mast cell activation, as judged by the extracellular release of tryptase in hanging mark’s tissues. Again, IL-15 can move up to complement the CD 15-based determination of ligature marks vitality with the accuracy needed for forensic purposes.

**Figure 7:**
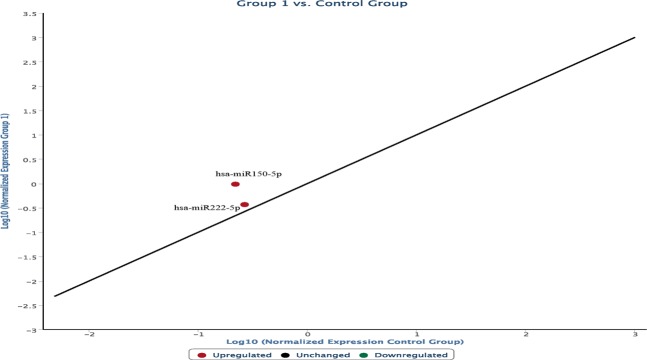
The results obtained showed an overexpression of miR222-5p and miR150-5p, looking for a possible correlation between miRNA expression and immunohistochemical findings reported in ligature marks, such as the positivity for tryptase, IL-15 and CD15. miR222-5p is involved in the activation of mast-cells, and miR150-5p is altered in neutrophils after traumatic tissue events.

The results obtained showed an overexpression of these miRNAs in both cases, although this was not statistically significant. (Fig. 7).

Finally, we further observed that a sample of non-injured skin from a subject of Asian origin and more precisely from Pakistan, compared to other healthy skin samples from non-Asian subjects, showed variations in the expression of miRNAs.

### Histological examination

**Figure 8:**
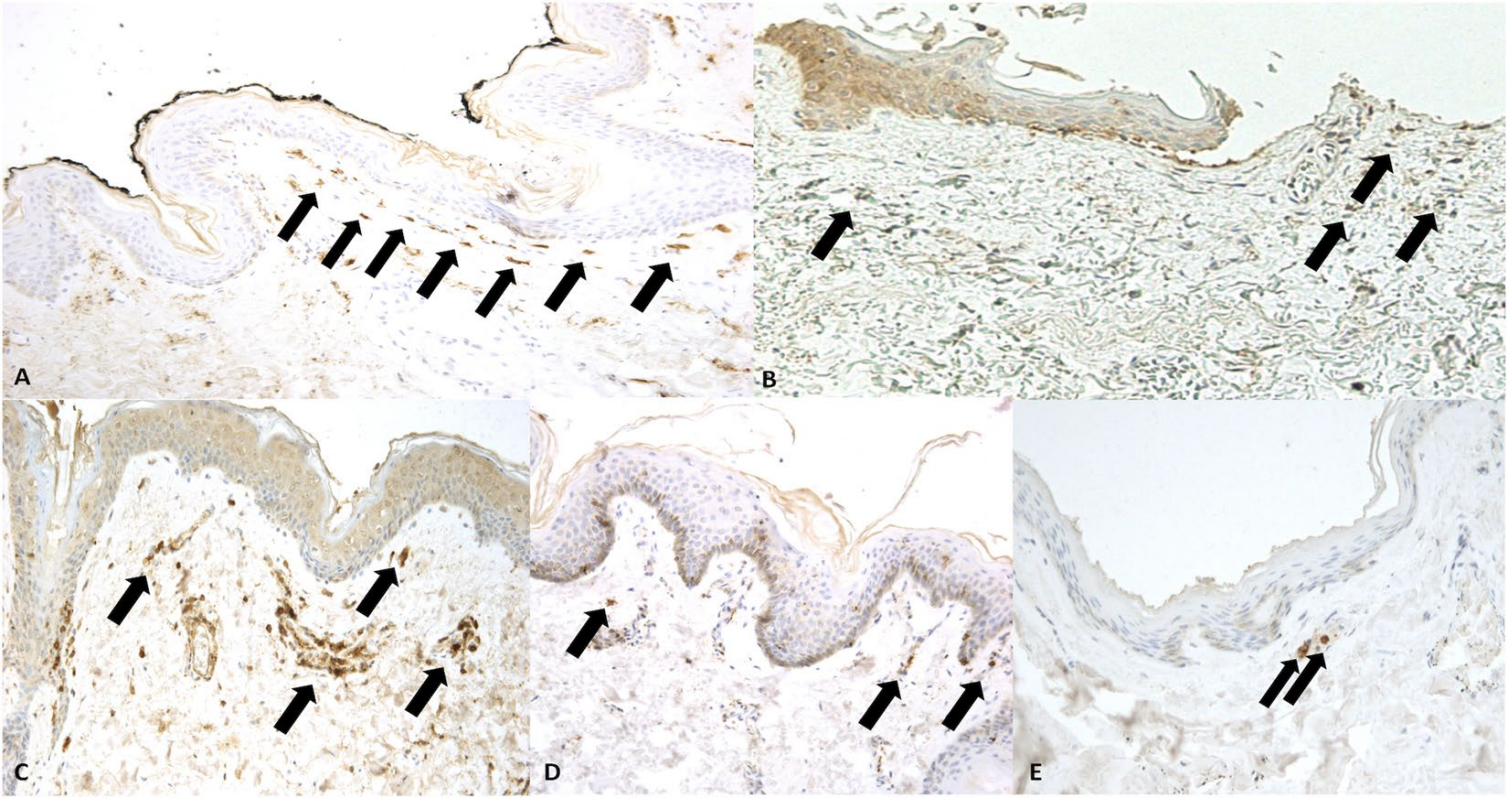
(**A**) Mast cells tagged by tryptase reaction. Mast cells appeared numerously (arrows), especially near the epidermis (x100). (**B**) Removal of epidermal fragments and intense mast cells positivity and halo around the cells (arrows) especially near the epidermis (x100). (**C**) CD15 reaction to demonstrate a few neutrophils near the vessels (arrows) (x100). (**D,E**) Randomly, sparse CD15 positivity (arrows) of intravital hanging (x100).

Immunohistochemistry showed a patchy dermal strong positivity of CD15 (++++), tryptase (++++), and IL-15 (++++) reaction in the marginal zones above and below the hanging marks. The microscopic observation of the samples showed the following structural differences: IL-15 was located around the dermal vessels and diffusely sparse in sub-dermal connective; CD15 and tryptase reactions were intense in dermal connective tissue. When CD15 reaction was present, IL-15 positivity was always observed to denote the earlier reaction referring to CD15, acting as a proinflammatory cytokine (Fig. 8).

The histological examination of control skin tissues was unremarkable.

## Discussion

The present study allowed a preliminary, mainly qualitative, assessment of miRNA expression in ligature marks in death by hanging, by comparing these skin samples with control skin samples.

The results obtained showed different microRNA skin expression profiles in ligature samples compared to control, non-injured samples.

As regards the upregulation of miRNAs linked to the inflammatory response in skin lesions (miR-146a-5p, miR146b-5p, miR125a-5p, miR125b-5p, miR155-5p and miR21-5p), in skin samples from ligature marks a statistically significant overexpression of miR146a-5p, miR125a-5p, miR125b-5p was detected.

The overexpression of the other miRNAs investigated can be explained based on the available evidence of their expression and their function at the tissue level.

miR133b, miR206, and miR1-3p are mainly found in skeletal muscle tissue since they are linked to the expression of inflammatory cytokines produced in degenerative myopathic conditions^28^.

miR128-3p, miR214-3p, and miR130a-3p, although not yet studied at skin level, have shown an anti-inflammatory action due to the inhibition of signal transduction pathways mediated by the transcription factor NFkB^29,30^. Similarly, miR122-5p inhibits NFkB nuclear translocation leading to a reduction in the expression of inflammatory cytokines^31^.

MiR92a-3p is involved in the intracellular regulation of signals activated by the toll-like receptor (TLR); therefore it could have a regulatory role in the inflammatory response^32^.

In conclusion, among the several hyperexpressed miRNAs found in skin samples from ligature marks, only miR125a-5p and miR125b-5p are today recognized as regulating molecules of the inflammatory response at skin level.

However, also miR214a-3p, miR128-3p, miR130a-3p and miR122-5p are implicated in the regulation of inflammatory response, especially with the inhibitory action of signal transduction pathways with pro-inflammatory function mediated by the transcription factor NFkB. In the same way, the miR92a-3p acts as a regulatory molecule that performs inhibitory activity on signal transduction pathways mediated by TLRs.

The difference in expression of the miRNAs of inflammatory cutaneous lesions (such as psoriasis, scleroderma, and dermatomyositis) compared to the skin of the ligature marks could hypothetically be traced back to the heterogeneous timing with which the former is produced^33–35^.

The lack of significant differences between frozen and formalin-frozen samples allows us to affirm the full applicability of the analysis method also to the formalin-fixed samples that are more commonly available to forensic pathologists.

Ultimately, it seems appropriate to highlight the difference in the expression of miRNAs in healthy skin in subjects of different ethnicity. This aspect implies the need to extend the series to reduce the influence of ethnic differences on the expression profiles of the obtained miRNAs.

## Conclusion

Conclusively, the results obtained showed an increase in the expression of miRNAs recognized as regulators of the inflammatory response in skin lesions such as miR125a-5p and miR125b-5p. Furthermore, overexpression of additional miRNAs – miR214a-3p, miR128-3p, miR130a-3p, miR122-5p and miR92a-3p – with anti-inflammatory activity was highlighted; however, it was possible to document a statistical significance compared with control skin samples only for miR214a-3p, miR130a-3p and miR92a-3p.

These data confirm that miRNA expression in traumatic cutaneous insult is to be referred to an act of regulation of the inflammatory phase aimed at inhibiting the intracellular signals activated by the production of inflammatory cytokines, even in cases of lesions that develop in a very short time, of the order of a few minutes.

The miRNA panel highlighted in the present study appears to be worthy of further study about the regulatory activity in the very early inflammatory response induced by traumatic insult. Furthermore, the upregulation of miR222-3p and miR150-5p, respectively related to mast-cell activation and neutrophils after the application of exogenous stimuli, also of a traumatic nature, supports the immunohistochemical data previously reported^12,24^. By comparing the difference in the expression of miRNAs in the lesions examined with those expressed in skin lesions originating in different periods, these biomarkers could become useful for the chronological diagnosis of the lesions, integrating the data relative to the expression of the miRNA with those shown by the results of histological and immunohistochemical investigations.

Our data suggest different miRNA expression profiles in skin samples with hanging ligature marks compared to normal control group samples, in accordance with previous studies demonstrating the key role of miRNAs in inflammatory response. Further investigation with a larger sample size should help validate these findings, focusing attention also on the possible difference in the expression of miRNAs related to the ethnicity of the subjects.

## Materials and Methods

### Samples

Our study was divided into two phases. In the first, we analyzed skin samples taken during autopsy and then frozen immediately. In the second phase, we tried to validate the results obtained from paraffin-embedded skin samples to verify the applicability of the miRNA investigation also to the autopsy samples routinely preserved with this method in our institutions.

To prevent possible influences in miRNA expression levels due to pathologies or diseases, all the subjects chosen were in an apparently good state of health before death. All samples were anonymized upon collection and discarded after use.

Specimens (hanging marks and control skin), corresponding to skin cross-sections of 1.5 to 4.0 cm, were collected during medico-legal autopsies. A total of 36 skin samples from ligature marks and 28 samples from non-injured skin of subjects who had died by suicidal hanging were analyzed. Only bodies free of post-mortem changes were selected and according to Italian Law 582/1994, about the method of assessment and certification of death, was performed EKG for 20 minutes to certificate death as soon as possible, so all the skin samples were collected within 12-24 hours after death.

Within one hour of excision, tissue samples were immersed in RNAlater® stabilization reagent solution (Qiagen®) and stored at −80 °C until further processing. Samples were then allowed to thaw on ice overnight and approximately 100 mg per sample were subjected to nucleic acid extraction.

To further assess the possible effects of degradation on miRNA profiling success, 20 skin samples from hanging ligature marks, formalin-fixed and paraffin-embedded (FFPE) before use, were collected for the study.

### RNA extraction and quantification

To remove ambient RNases, all surfaces and devices utilized during the extraction procedure were thoroughly cleaned using RNaseZap - RNase Decontamination Solution (Thermo Scientific®). Furthermore, only RNase‐free reagents, plastic consumables, and instruments were used. Depending on features of the samples (fresh or FFPE tissues) specimens were extracted using the miRNeasy Mini kit (Qiagen®) and miRNeasy FFPE kit (Qiagen®) according to the manufacturer’s protocols. In both cases, treatment with DNase‐I was included in the protocol to remove potential genomic DNA traces. The quantity of RNA was assessed using the BioPhotometer UV/Vis spectrophotometer (Eppendorf®). Extracts were immediately stored at −20 °C until further use.

### Selection of target genes

The selection of organ tissue-specific candidate markers focused on molecules involved during wound healing^36,37^.

Because hanging causes the death of the subject in a few minutes^38,39^, candidate target genes were represented by those expressed in the earliest steps of the inflammatory skin process in the ligature marks^40^.

Identified markers expression profiling was accomplished using miScript® miRNA PCR Array - Human Cell differentiation & Development (Qiagen®).

**Figure 9:**
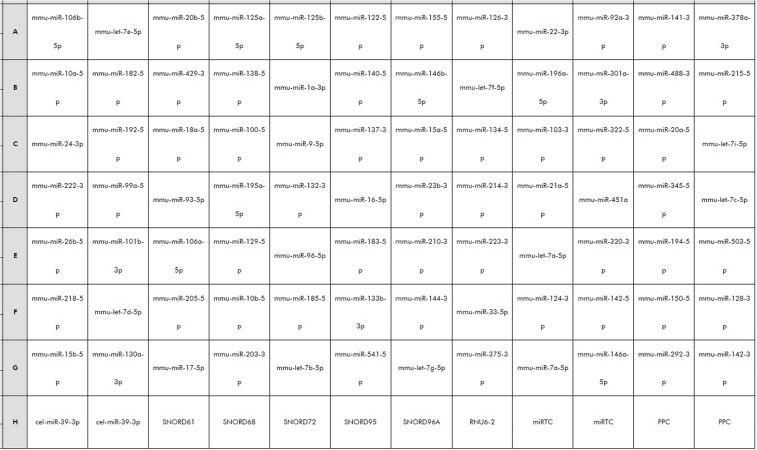
Pathway-Focused miRNA PCR array layout. Wells A1 to G12 (1-84) each contain a miScript primer assay for a pathway related mature miRNA. Wells H1 and H2 contain replicate C. elegans miR-39 (Ce) assays that can be used as an alternative normalizer for array data. Wells H3 to H8 each contain an assay for a different snoRNA/snRNA that can be used as a normalization control for the array data. Wells H9 and H10 contain replicate miRTC assay used as assessment of reverse transcription performance. Finally, wells H11 and H12 contain replicate positive PCR controls (PPC).

Figure 9 shows miRNA target molecules and array layout.

### Complementary DNA synthesis

Multiplexed cDNA synthesis was performed using the miScript II RT kit® (Qiagen®), in a volume of 20 μL and according to manufacturer’s instructions for creating a custom reverse transcription (RT) pool.

Reactions were performed on a Veriti™ 96-Well Fast Thermal Cycler (Thermo Scientific®) with the following cycling conditions: 60 °C for 37 min and 95 °C for 5 min to inactivate miScript Reverse transcriptase. Extraction negative and H2O controls were performed on a sample basis and RT(−)-controls were set up to control for potential contamination with genomic DNA.

After the reaction, cDNAs were placed at −20 °C until further use. Before RT-PCR, cDNAs were diluted using 180 μL of RNase-free water.

### Real-time quantitative polymerase chain reaction

cDNAs prepared in a reverse-transcription reaction served as a template for RT-PCR analysis using miScript® miRNA PCR Array - Human Cell differentiation & Development (Qiagen®), which contains miRNA-specific miScript primer assays) and miScript SYBR Green kit (Qiagen®), which contains miScript universal primer (reverse primer), according to the manufacturer’s protocol.

For mature miRNA expression and profiling, 100 μL of total RNA added to the reaction mix containing miScript universal primer, QuantiTect SYBR Green PCR master mix, and RNase-free water, in a final volume of 2750 μL.

Arrays were designed in a 96-well format; the final 12 wells of each array contained reaction controls as follows: 5 snoRNAs (SNORD61, SNORD68, SNORD72, SNORD95, and SNORD96A) and snRNA RNU6B (RNU6-2) using for normalization and allowing from different experiments and samples to be compared directly. Normalization corrects for factors, i.e., the quantity of RNA input, RNA degradation or presence of inhibitors, and differences in sample handling, that could otherwise lead to inaccurate quantification.

Six wells were performed:

- Two miRTC wells containing reverse transcription controls, assessing the performance of the reverse transcription reaction. These controls monitor for any variables that may inhibit the reverse transcription reaction.
- Two positive PCR control (PPC) wells containing a predisposed artificial DNA sequence and the assay that detects it. These controls monitor for any variables that may inhibit the miScript® miRNA PCR Array reaction.
- two wells, named ‘Ce’, containing C. Elegans miR-39 primer assay useful for the normalization of RT-PCR results for endogenous reference RNAs (SNORD61, SNORD68, SNORD72, SNORD95, SNORD96A, and RNU6-2).

All assays were run on an AB 7300 Real-Time PCR equipment (Thermo Scientific®) and thermal cycling conditions were as follows: initial hold for 15 min at 95 °C, followed by 40 cycles of 94 °C for 15 s, 55 °C for 30 sec and 70 °C for 40 sec were carried out.

To compare results, all data analyses were accomplished by setting up the same values of baseline and threshold as recommended by miScript® miRNA PCR Array handbook.

Once the Ct values were exported, quantification was performed using the ΔΔCt method of relative quantification and interpretation of control wells using the miScript miRNA PCR array web-based software (Qiagen®) following the manufacturer’s instructions.

### Histological examination

In all cases of hanging, sections of skin were removed from the neck at the site of the greater depth of the marks. In control cases skin samples were taken from the anterior face of the neck.

A routine microscopic histopathological study was performed using hematoxylin-eosin (H&E) staining. In addition, immunohistochemical investigation of skin samples was performed utilizing antibodies anti-tryptase, IL-15, CD 15.

We used 4μm thick paraffin-embedded sections, mounted on slides covered with 3, amminopropyltriethoxysilane (Fluka, Buchs, Switzerland). The sections in paraffin were re-hydrated and incubated for 20 minutes in methanol containing 10% of H2O2 to block endogenous peroxidases. The sections were pre-treated to facilitate antigen retrieval and to increase membrane permeability to antibodies and then incubated with the primary antibody. Tryptase: 5 min Proteolytic Enzyme (Dako, Copenhagen, Denmark), 20 °C 120 min, 20 °C 1:1000. IL-15: (R&D Systems, Inc. Minneapolis, USA) boiling in 0.25 mM EDTA buffer. 120 min, 20 °C 1:100. CD 15: (DAKO, Copenhagen, Denmark) boiling in 0.25 mM EDTA buffer. 120 min, 20 °C 1:50. The detection system utilized was the LSAB + kit (Dako, Copenhagen, Denmark), a refined avidin–biotin technique in which a biotinylated secondary antibody reacts with several peroxidise conjugated streptavidin molecules. The positive reaction was visualized by 3,3-diaminobenzidine (DAB) peroxidation, according to standard methods. The sections were counterstained with Mayer’s hematoxylin, dehydrated, covers-lipped and observed in a Leica DM4000B optical microscope (Leica, Cambridge, UK) connected to a computerized system with photo camera (DC 480 Leica, Cambridge, UK).

A semi-quantitative evaluation of the immunohistochemical findings by two different investigators (MN, AM) without prior knowledge was performed; all measurements were done at the same magnification of image (x10) and the following gradation of the immunohistochemical reaction was used in the scale 0-4, as follows:

- (0): not expressed,
- (+): isolated and disseminated expression,
- (++): expression in groups or widespread foci,
- (+++): widespread expression,
- (++++): massive and diffuse positivity.

### Statistical analysis

Semi-quantitative evaluation of the immunohistochemical findings and gradation of the immunohistochemical reaction were described with an ordinal scale and the median value reported. Analysis of variance for the non-parametric data was performed using Kruskal-Wallis test. When differences were found to be significant, analysis between the unmatched groups were elucidated with a Dunn’s Multiple Comparison post hoc test. Significance level was set to 5% (SPSS ver. 16.01 for Windows – SPSS Inc., Chicago USA).

### Ethical approval and informed consent

Data processing complies with the general authorization for scientific research purposes granted by the Italian Data Protection Authority (1 March 2012 as published in Italy’s Official Journal no. 72 dated 26 March 2012) since the data do not entail any significant personalized impact on data subjects. Approval by an institutional and/or licensing committee is not required since experimental protocols are not applied in the study. All cases are judicial and come from autopsies ordered by local prosecutors to clarify the exact cause of death.
